# Amphotericin B colloidal dispersion: an effective drug for the treatment of mucormycosis in China

**DOI:** 10.3389/fcimb.2023.1147624

**Published:** 2023-05-17

**Authors:** Juntao Liu, Xiaoxu Ma

**Affiliations:** ^1^ Department of Thoracic Surgery, The First Affiliated Hospital of Zhengzhou University, Zhengzhou, Henan, China; ^2^ Department of Respiration, The First Affiliated Hospital of Zhengzhou University, Zhengzhou, Henan, China

**Keywords:** amphotericin B colloidal dispersion, hematological malignancies, mucormycosis, efficacy, safety

## Abstract

**Objective:**

Mucormycosis has emerged as an increasingly important cause of morbidity and mortality in immunocompromised patients, but the effective drugs for the treatment are limited. Hence, the study aimed to summarize the characteristics of mucormycosis in patients with hematological malignancies, and investigate the efficacy and safety of Amphotericin B Colloidal Dispersion (ABCD) in treating mucormycosis.

**Methods:**

In this study, patients with mucormycosis complicated by hematological malignancies who received ABCD at the First Affiliated Hospital of Zhengzhou University from April 2021 to May 2022 were retrospectively enrolled. The clinical data of the enrolled patients were collected, and then, the drug response at 2 weeks, 4 weeks, and the end of treatment; the survival rate at 4, 8, and 12 weeks; and the laboratory-related indicators and adverse events (AEs) associated with ABCD were evaluated.

**Results:**

In total, 9 patients with mucormycosis complicated by hematological malignancies were enrolled. The main symptoms were fever, cough, and chest pain. In addition, reversed halo signs (RHS) were found on chest CTs. The responses to ABCD at 2 weeks, 4 weeks, and the end of treatment were 100% (9/9), 77.8% (7/9), and 77.8% (7/9), respectively. The survival rates of the patients at 4, 8, and 12 weeks were 77.8% (7/9), 66.7% (6/9), and 66.7% (6/9), respectively. Among laboratory-related indicators, white blood cell (WBC) counts were significantly increased from baseline after 1 and 2 weeks of ABCD treatment (*P*<0.05), whereas neutrophil counts were only increased significantly from baseline at 2 weeks post-treatment (*P*<0.05). The most common AEs were infusion-related AEs manifesting as fever, chills, and pruritus. Moreover, none of the patients suffered from renal injury once again.

**Conclusion:**

ABCD is a promising treatment strategy for patients with mucormycosis complicated by hematologic malignancies, showing remarkable efficacy and safety.

## Introduction

Mucormycosis is a rare disease with high morbidity and mortality, which is difficult to diagnose (Cornely et al., 2019). Hematological malignancies are the main underlying diseases associated with mucormycosis. The available data suggest that mucormycosis has the highest mortality rate among all types of fungal infections (Bretagne et al., 2022), whereas hematologic malignancies ranked the first in the mortality of all primary diseases among mucormycosis patients (Roden et al., 2005; Muthu et al., 2021). Mucormycosis has thus become one of the most important causes of death in patients with hematologic malignancies. Although surgical debridement combined with antifungal drug therapy can improve the survival rate of patients with mucormycosis, compared to that with antifungal drugs alone, unfortunately, many patients with hematologic malignancies are not eligible for surgery owing to susceptibility to infection and bleeding. Therefore, systemic antifungal drug therapy remains the most important treatment for many patients with hematologic malignancies (Muthu et al., 2021). In addition, compared to that with early treatment, delaying amphotericin B (AmB)-based first-line therapy was shown to lead to a 2-fold increase in the mortality rate at 12 weeks after diagnosis (Chamilos et al., 2008). Thus, timely and effective treatment is critical for improving prognosis.

The main drugs recommended for this are liposomal amphotericin B (L-AmB), isavuconazole, and posaconazole based on the revised consensus definitions of IFD from EORTC/MSGERC. L-AmB is preferred when all three drugs are available (Cornely et al., 2019). Unfortunately, L-AmB made in China is different from L-AmB in other countries, and there is no significant improvement in nephrotoxicity compared with AmB (Caillot et al., 2018), and even worse, few patients can afford isavuconazole and posaconazole because of their high price, and they have not been included in China’s medical insurance system. Consequently, AmB and Amphotericin B Colloidal Dispersion (ABCD) are the main drugs presently used to treat mucormycosis in China. Sodium deoxycholate has been used as a co-solvent of the traditional AmB, but this readily causes renal insufficiency and severe hypokalemia, severely limiting the dosage and duration of medication and affecting its antifungal effect (Ullmann et al., 2006). Moreover, it usually takes months to receive antifungal treatment for patients with mucormycosis complicated by hematologic malignancies. Hence, AmB is far from meeting clinical demands. ABCD is a disc-shaped colloidal nanoparticle dispersion formed with AmB and sodium cholesterol-sulfate at a molar ratio of 1:1. Sodium cholesterol sulfate can combine with AmB, thereby reducing the combination of AmB with cholesterol in the human cell membrane (Herbrecht et al., 2001). In addition, ABCD can be quickly absorbed by reticuloendothelial system organs, such as the liver, spleen, and lung, after entering the blood, thus avoiding damage to the renal tubules; therefore, nephrotoxicity is low (Oppenheim et al., 1995). Adverse reactions to ABCD mainly comprise an infusion reaction (White et al., 1998). Since ABCD (CSPC Ouyi Pharmaceutical Group Co., Ltd.) got successful marketing authorisation by the National Medical Products Administration in China on March 30, 2021, as a polyene drug with minimal renal toxicity, it has been increasingly used for antifungal therapy. However, to our knowledge, there are no relevant research reports on the efficacy and safety of ABCD in China for patients with mucormycosis complicated by hematologic malignancies.

Therefore, for the first time, we summarized the clinical characteristics of 9 patients with mucormycosis complicated by hematologic malignancies and treated with ABCD. Moreover, we assessed the efficacy and safety of ABCD for the treatment of mucormycosis, expecting to provide evidence for clinical application.

## Patients and methods

### Study design

In this study, data of patients diagnosed with mucormycosis complicated by hematologic malignancies, who had received ABCD treatment for more than 7 days with complete laboratory data, at the First Affiliated Hospital of Zhengzhou University from April 2021 to May 2022 were retrospectively collected. The mucormycosis diagnosis was in accordance with the revised consensus definitions of IFD from EORTC/MSGERC (Cornely et al., 2019), and the patients included were classified into three categories, “proven” “probable” and “possible”. Specifically, 2 cases were proven, 6 cases were probable, and 1 case was possible. All included patients were diagnosed by a respiratory specialist, a hematologist, and a radiologist. This study was approved by the Ethics Committee of the First Affiliated Hospital of Zhengzhou University (2021-KY-0286). The requirement for informed consent was waived owing to the retrospective nature of the study. The hospital electronic database was searched to collect clinical data of patients, including demographic, primary morbidity, clinical manifestations, imaging features, laboratory examination, diagnosis processes, treatment measures, and adverse events (AEs).

### Evaluation of clinical response

The response to ABCD at 2 weeks, 4 weeks, and the end of treatment and the survival rate at 4, 8, and 12 weeks post-treatment were evaluated according to the *2008 Mycoses Study Group and European Organization for Research and Treatment of Cancer Consensus Criteria* (De Pauw et al., 2008). Based on the basis of a composite of clinical, radiological, and mycological criteria, the general criteria for global responses to antifungal therapy were classified as follows: 1) complete response (CR), survival within the prespecified period of observation, resolution of all attributable symptoms and signs of disease and radiological abnormalities, and mycological evidence of eradication of disease; 2) partial response (PR), survival within the prespecified period of observation, improvement in attributable symptoms and signs of disease and radiological abnormalities, and evidence of clearance of cultures or reduction of fungal burden. As assessed by a quantitative and validated laboratory marker; 3) stable response, survival within the prespecified period of observation and minor or no improvement in fungal disease, but no evidence of progression, as determined on the basis of a composite of clinical, radiological, and mycological criteria; 4) progression of fungal disease, evidence of progressive fungal disease based on a composite of clinical, radiological, and mycological criteria (Chamilos et al., 2008); death, death during the prespecified period of evaluation, regardless of attribution. CR and PR were defined as success, whereas the remaining states were considered failure.

Laboratory indicators, including routine blood tests, liver function, renal function, and electrolytes, were assessed. Drug safety was evaluated based on the AEs recorded according to the National Cancer Institute Common Terminology Criteria for Adverse Events (CTCAE, version 5.0). Clinical AEs caused by ABCD were defined as parameters that changed from normal to abnormal or aggravated compared to those at baseline, which occurred during treatment with the drugs.

### Statistical analysis

All data analyses were performed using SPSS version 25.0 (SPSS Inc. IBM Corp). Continuous data were tested for normality using the Shapiro-Wilk method. If the normality criteria were satisfied, data were described as the mean ± standard deviation, and the comparisons were performed using paired-samples Student’s *t*-tests. Otherwise, the median and interquartile ranges (IQR) were used. The categorical data were expressed as percentages (%) and frequencies. Missing values were not included in the percentage calculations unless otherwise noted. Effect sizes were reported with 95% confidence intervals. Statistical significance was defined as *P*<0.05 for all analyses.

## Results

### Baseline characteristics

**Figure 1:**
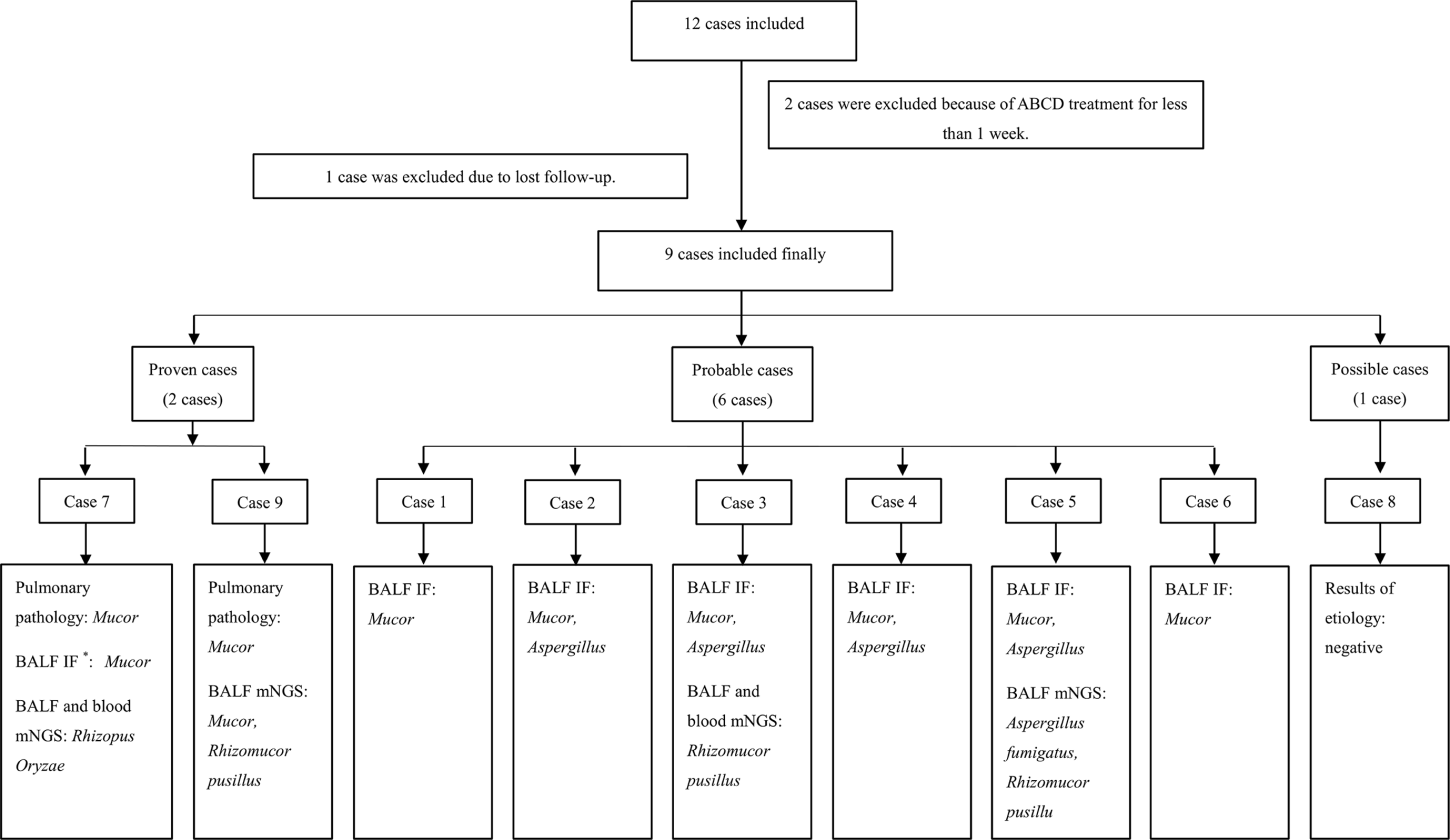
Flow chart of patients’ inclusion and diagnosis. ^*^BALF immunofluorescen staining of *Mucor* and *Aspergillus*: *Mucor*, broad undivided, right-angled branching hyphae; *Aspergillus*, widely septate, branching hyphae at acute angles. ABCD, Amphotericin B Colloidal Dispersion; BALF, bronchoalveolar lavage fluid; IF, immunofluorescen staining; mNGS, metagenomic next generation sequencing.

There were 12 patients diagnosed with hematological malignancies complicated by mucormycosis, of which, 2 were excluded because they were treated for fewer than 7 days with ABCD and one was excluded as they were lost to follow-up. A total of 9 cases were included in the analysis (**Figure 1**). Among the 9 patients, 6 were males and 3 were females, with a median age of 40 years. The underlying diseases were acute myeloid leukemia (6 cases), acute lymphoblastic leukemia (2 cases) and T-lymphoblastic lymphoma (1 case). According to the revised consensus definitions of IFD from EORTC/MSGERC (Cornely et al., 2019), 2 were proven cases, 6 were probable cases and 1 was possible case among the 9 patients. Additionally, 6 cases were pulmonary mucormycosis and 3 cases were disseminated mucormycosis (pulmonary and cerebral mucormycosis). Fever, cough, and chest pain were the main symptoms. All cases had reversed halo signs (RHS) on chest CT, of which, Case 3 had multiple nodules in the lungs and Cases 6 and 7 simultaneously had pleural effusion. Before the administration of ABCD, 7 patients received AmB and posaconazole combined antifungal therapy, 1 patient received posaconazole combined with voriconazole therapy, and 1 patient received voriconazole as antifungal monotherapy. However, all patients had varying degrees of renal damage, nausea, and vomiting. Among the 9 cases, Case 3 received ABCD as the first-line therapy, Case 9 was treated with ABCD as an alternative treatment, and the remaining 7 cases received ABCD as salvage treatment. Detailed information was shown in **Table 1**.

**Table 1:** Demographic and clinical characteristics at baseline.

Variables	Case 1	Case 2	Case 3	Case 4	Case 5	Case 6	Case 7	Case 8	Case 9
Age, (years)	46	40	30	20	51	24	15	67	47
Gender	Male	Male	Male	Female	Male	Male	Male	Female	Female
Body weight, (kg)	70.0	74.0	60.0	46.0	50.0	42.5	48.0	57.0	55.0
ECOG	3	2	2	2	4	3	3	3	3
Underlying disease	AML	AML	AML	ALL	T-LBL	AML	AML	ALL	AML
Treatment of underlying diseases	Chemotherapy	Chemotherapy	Chemotherapy	Chemotherapy	Chemotherapy	Chemotherapy	Chemotherapy	CAR-T	Chemotherapy
Symptoms									
Fever	Yes	Yes	Yes	Yes	Yes	Yes	Yes	Yes	Yes
Cough	Yes	Yes	Yes	Yes	Yes	Yes	Yes	Yes	Yes
Chest pain	Yes	Yes	Yes	Yes	Yes	Yes	Yes	No	Yes
Glucocorticoid treatment history	DXM	DXM	No	DXM	DXM	DXM	DXM	MP	DXM
Previous antifungal agents	Posaconazole, AmB	Posaconazole, VRC, AmB	VRC	Posaconazole, VRC	Posaconazole, VRC, AmB	Posaconazole, AmB	Posaconazole, VRC, AmB	Posaconazole, VRC, AmB	Posaconazole, VRC, AmB
Previous stage of antifungal treatment	Diagnosis-driven treatment	Diagnosis-driven treatment	Diagnosis-driven treatment	Diagnosis-driven treatment	Diagnosis-driven treatment	Diagnosis-driven treatment	Targeted treatment	Diagnosis-driven treatment	Targeted treatment
Previous adverse events									
Renal injury	Yes	Yes	Yes	Yes	Yes	Yes	Yes	Yes	Yes
Vomiting	Yes	Yes	Yes	Yes	Yes	Yes	Yes	Yes	Yes
Mycological evidence									
BALF IF	*Mucor*	*Mucor, Aspergillus*	*Mucor, Aspergillus*	*Mucor, Aspergillus*	*Aspergillus, Mucor*	*Mucor*	*Mucor*	NA	NA
BALF mNGS	NA	NA	*Rhizomucor pusillus*	NA	*Aspergillus fumigatus, Rhizomucor pusillus*	NA	*Rhizopus Oryzae*	NA	*Mucor, Rhizomucor pusillus*
Blood mNGS	NA	NA	*Rhizomucor pusillus*	NA	NA	NA	*Rhizopus Oryzae*	NA	NA
Sputum fungal culture	NA	NA	NA	NA	NA	NA	NA	NA	NA
Pulmonary pathology	NA	NA	NA	NA	NA	NA	*Mucor*	NA	*Mucor*
CT signs	RHS	RHS	RHS, Multiple nodules	RHS	RHS	RHS, Hydrothorax	RHS, Hydrothorax	RHS	RHS
Classification of diagnosis	Probable	Probable	Probable	Probable	Probable	Probable	Proven	Possible	Proven
Effect of previous antifungal drugs	Intolerant	Intolerant	Intolerant	Intolerant	Ineffective	Intolerant	Ineffective	Intolerant	Intolerant
ABCD treatment phase	Salvage treatment	Salvage treatment	First-line therapy	Salvage treatment	Salvage treatment	Salvage treatment	Salvage treatment	Salvage treatment	Alternative treatment

ECOG, Eastern Cooperative Oncology Group; AML, acute myeloid leukemia; ALL, acute lymphoblastic leukemia; T-LBL, T-lymphoblastic lymphoma; CAR-T, chimeric antigen receptor T-Cell immunotherapy; BALF, bronchoalveolar lavage fluid; IF, immunofluorescence; mNGS, metagenomic next generation sequencing; DXM, dexamethasone; MP, methylprednisolone; VRC, voriconazole; RHS, reversed halo signs; NA, not available; ABCD, Amphotericin B Colloidal Dispersion.

Bronchoscopy was performed in all cases. Cases 7 and 9 were proven by biopsy using fiberoptic bronchoscopy, which indicated *Mucor* infection. In the probable cases, *Mucor* filaments were detected via immunofluorescence in the bronchoalveolar lavage fluid (BALF), including 4 cases of both *Aspergillus* and *Mucor* filaments. Case 8 was the only possible case in which mycological evidence were all negative in the present study. Furthermore, among all the 9 cases, BALF metagenomic next generation sequencing (mNGS) for Cases 3, 7, 5, and 9 suggested *Mucor*, including 1 case (Case 5) suggesting mixed infection of *Aspergillus fumigatus* and *Rhizomucor pusillus*. Additionally, peripheral blood mNGS and BALF mNGS were used for Cases 3 and 7 simultaneously, and the results were consistent between these two methods. Sputum fungal culture was negative in all cases. Detailed information was shown in **Table 1**.

As shown in **Table 2**, before ABCD treatment, 8 of the 9 patients had mild-to-moderate anemia, 6 had severe neutropenia when diagnosed with mucormycosis, and 3 had extremely severe thrombocytopenia. In terms of renal function, 2 patients (Cases 2 and 7) had elevated urea nitrogen. Regarding liver function, the elevated levels of total, direct, and indirect bilirubin were observed in Case 7. Glutamyl transpeptidase (GGT) levels were significantly increased in 2 cases (Cases 2 and 5) and slightly increased in Case 9. The levels of alanine aminotransferase (ALT) and alkaline phosphatase (ALP) were significantly increased in 2 cases (Cases 2 and 5), and the levels of aspartate aminotransferase (AST) were significantly increased in Case 2.

**Table 2:** Laboratory indicators at baseline.

Variables	Case 1	Case 2	Case 3	Case 4	Case 5	Case 6	Case 7	Case 8	Case 9	
WBC, (10^9/L)	6.04	0.7	0.24	0.27	1	10.87	0.16	0.07	5.89	
RBC, (10^12/L)	2.54	2.5	2.42	2.86	3.05	2.58	2.7	3.51	3.08	
HB, (g/L)	80.2	73	71	89	86	79.4	79	110	96	
PLT, (10^9/L)	115	6	63	36	3	63	10	2	243	
Neutrophil count, (10^9/L)	4.98	0.15	0.07	0.04	0.23	8.07	0.02	0.01	4.24	
Lymphocyte count, (10^9/L)	0.35	0.22	0.15	0.3	0.17	0.79	0.13	0.05	0.92	
BUN, (mmol/L)	2.7	14.8	3.8	1.9	6.5	/	12.8	6.6	2.1	
Scr, (μmol/L)	57	/	42	34	19	/	59	38	34	
TBIL, (μmol/L)	10.01	/	5.9	13.1	12.6	/	45.1	/	5.2	
DBIL, (μmol/L)	3.8	18.9	2.6	10.7	9.3	/	22.6	/	2.6	
IBIL, (μmol/L)	6.3	6	3.3	2.4	3.3	/	22.5	/	2.6	
GGT, (U/L)	16	710	45	33	289	/	34	/	70	
ALT, (U/L)	25	122	23	43	132	/	18	20	17	
AST, (U/L)	16	100	15	76	21	/	9	8	28	
ALP, (U/L)	76	291	79	158	498	/	113	50	89	
TP, (g/L)	58.9	58.4	59.2	36.2	44.5	50	60.7	42.8	61.8

WBC, white blood cells; RBC, red blood cells; HB, hemoglobin; PLT, platelet; BUN, blood urea nitrogen; Scr, serum creatinine; TBIL, total bilirubin; DBIL, direct bilirubin; IBIL, indirect bilirubin; GGT, glutamyltranspeptidase; ALT, alanine aminotransferase; AST, aspartate aminotransferase; ALP, alkaline phosphatase; TP, total protein. /, data not available.

### ABCD therapy

In all included cases, ABCD was infused via a peripherally inserted central venous catheter (PICC) for 8-10 h. The dose on the first day was 50 mg, which was increased to 150-200 mg within 2-4 days. The median duration of treatment was 33 (13-88) days. Taken together, the accumulated dose for the nine patients was 5800 (2200-16900) mg, and the mean dose was 3.33 (1.96-4.35) mg/kg/d. Among the nine cases, four were treated with posaconazole enteric-coated tablets or suspension maintenance after effective ABCD treatment, and 5 discontinued with ABCD for economic reasons (2 cases; Cases 1 and 6), primary disease recurrence (2 cases; Cases 2 and 5), and death resulting from massive hemoptysis caused by IFD (1 case; Case 7) (**Table 3**). The administration of ABCD in each patient is shown in **Supplementary Table 1**.

**Table 3:** Amphotericin B colloidal dispersion (ABCD) treatment.

Variables, (no. (%)/Median (IQR))	
Mode of infusion, (PICC)	9 (100.00)
Duration of infusion, (h)	8 (8-10)
Initial dose, (mg)	50 (50-50)
Ascending time to maximum dose, (days)	5 (3-31)
Maintenance of maximum dose, (mg)	200 (150-200)
Treatment duration, (days)	38 (13-88)
Cumulative dose, (mg)	5800 (2200-16900)
Mean dose, (mg/kg/d)	3.33 (1.96-4.35)
Reasons for termination of treatment	
Switch to oral medication	4 (44.44)
Treatment abandonment^*^	5 (55.56)

^*^there were five patients who discontinue ABCD treatment, including two cases for economic reasons, two cases because of the recurrence of primary disease, and one case of massive hemoptysis caused by fungal infection.

### Adjuvant therapy

All patients were treated with promethazine and dexamethasone prior to ABCD to prevent possible infusion-related AEs, which occurred in 8 cases after 0.5-1 h of ABCD treatment on the first day. After stopping the infusion of ABCD and giving methylprednisolone, all patient symptoms related to infusion-related AEs were relieved, and then continued to receive ABCD treatment.

After the infusion-related AEs, ALT, AST, total bilirubin (TBIL), direct bilirubin (DBIL), indirect bilirubin (IBIL), procalcitonin (PCT), and C-reactive protein (CRP) were elevated transitionally in 8 patients. Therefore, glutathione (GSH), magnesium isoglycyrrhizinate (MgIG), and other hepatoprotective drugs were administrated. Liver function in 6 patients returned to normal. In the absence of other new infections, PCT and CRP levels returned to pre-ABCD levels in approximately 7 days. All patients were treated with antifungal combination therapy via the oral administration of AmB atomization and posaconazole tablets or suspensions while using ABCD treatment. In addition, the posaconazole concentration for Case 1 was higher than 5.00 μg/mL, that for Case 6 was 0.61 μg/mL, and that for the other 7 cases was 1.00-3.00 μg/mL (**Table 4**).

**Table 4:** Adjuvant therapy.

Cases	Pretreatment	Maximal dose, (mg)		Combined antifungal drugs	Posaconazole serum concentration, (μg/mL)	Liver protection drugs
		DXM	Promethazine			
1	DXM; Promethazine	5	25	AmB; Posaconazole	>5.00	GSH
2	DXM; Promethazine	5	25	AmB; Posaconazole; Voriconazole	2.57	GSH; MgIG
3	DXM; Promethazine	5	25	AmB; Posaconazole	1.89	GSH
4	DXM; Promethazine	5	10	AmB; Posaconazole	2.43	GSH; MgIG
5	DXM; Promethazine	5	25	AmB; Posaconazole	2.24	GSH; MgIG; DG
6	DXM; Promethazine	5	25	AmB; Posaconazole; Voriconazole	0.61	GSH; MgIG
7	DXM; Promethazine	5	25	AmB; Posaconazole	2.25	GSH
8	DXM; Promethazine	5	10	Posaconazole	1.22	GSH; MgIG; Bicyclol
9	DXM; Promethazine	2	25	AmB; Posaconazole	1.96	None

DXM, dexamethasone; AmB, Amphotericin B; GSH, glutathione; MgIG, magnesium isoglycyrrhizinate; DG, diammonium glycyrrhizinate.

### Efficacy analysis

**Figure 2:**
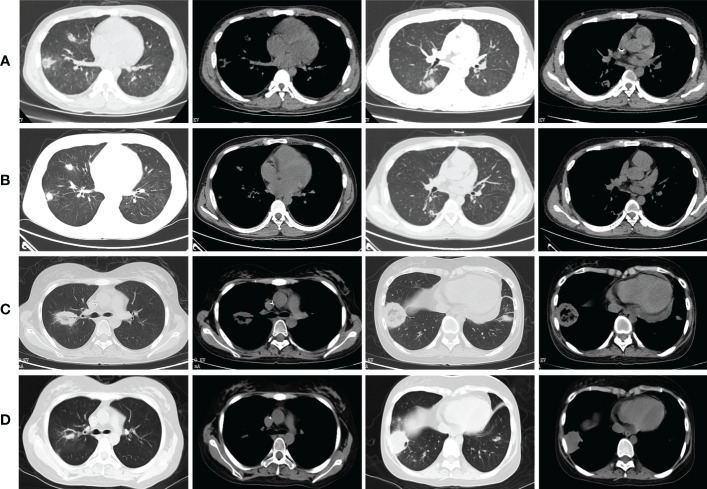
Findings of chest CT scans before and after ABCD treatment in cases 3 and 4. **(A)** Before ABCD treatment, multiple nodules and reversed halo signs were seen in both lungs; **(B)** After 6 weeks of ABCD treatment, both lung lesions were significantly reduced; **(C)** Before ABCD treatment, reversed halo signs were observed in the upper and lower lobes of the right lung; **(D)** After 6 weeks of ABCD treatment, the lesions were significantly reduced, and cavity was formed in the upper lobe of the right lung, while low-density necrosis and small cavity was found in the lesions in the lower lobe of the right lung.

As shown in **Table 5**, at 2 weeks of ABCD treatment, all 9 patients achieved PR with a drug response of 100% (9/9). At 4 weeks of treatment, 7 patients achieved PR, with a drug response of 77.8% (7/9). At the end of treatment, 6 patients achieved PR and 1 achieved CR (Case 3), with a drug response of 77.8% (7/9). **Figure 2** showed the findings of chest CT scans before and after ABCD treatment in cases 3 and 4. In Case 3, before ABCD treatment, multiple nodules and RHS were seen in both lungs (**Figure 2A**); after 6 weeks of ABCD treatment, both lung lesions were significantly reduced (**Figure 2B**). In Case 4, before ABCD treatment, RHS were observed in the upper and lower lobes of the right lung (**Figure 2C**); after 6 weeks of ABCD treatment, the lesions were significantly reduced, and cavity was formed in the upper lobe of the right lung, while low-density necrosis and small cavity was found in the lesions in the lower lobe of the right lung (**Figure 2D**).

**Table 5:** Efficacy indicators and end point evaluation.

Cases	ABCD response			ECOG		Fungal burden		Imaging remission		Prognosis		
	2 weeks	4 weeks	End of treatment	2 weeks	4 weeks	Negative or not	Time, (days)	2 weeks	4 weeks	Week 4	Week 8	Week 12
1	PR	PR	PR	2	2	Yes	8	Yes	Yes	Survival	Survival	Survival
2	PR	PR	PR	1	3	No	/	Yes	Yes	Survival	Death^#^	Death
3	PR	PR	CR	1	1	No	/	Yes	Yes	Survival	Survival	Survival
4	PR	PR	PR	1	1	No	/	Yes	Yes	Survival	Survival	Survival
5	PR	/^*^	/	4	/	No	/	Yes	/	Death^#^	Death	Death
6	PR	PR	PR	2	2	No	/	Yes	Yes	Survival	Survival	Survival
7	PR	/^*^	/	1	/	Yes	17	Yes	/	Death^#^	Death	Death
8	PR	PR	PR	3	2	Yes	33	Yes	Yes	Survival	Survival	Survival
9	PR	PR	PR	1	1	Yes	23	Yes	Yes	Survival	Survival	Survival

^*^the total treatment duration for Case 5 and Case 7 was less than 4 weeks. ^#^Case 7 died of massive hemoptysis caused by mucormycosis, and Case 5 died due to recurrence of the primary disease after less than 4 weeks of treatment; Case 2 died due to co-bacterial infection between 4 and 8 weeks. ECOG, Eastern Cooperative Oncology Group; PR, partial response; CR, complete response. /, data not available.

Survival rates of patients at 4, 8, and 12 weeks were 77.8% (7/9), 66.7% (6/9), and 66.7% (6/9), respectively. Notably, Case 5 and Case 7 both died within 4 weeks after ABCD treatment due to recurrence of underlying disease and massive hemoptysis caused by IFD, respectively. Case 2 died resulted from co-bacterial infection between 4 and 8 weeks of post-treatment.

### Laboratory data analysis

As shown in **Table 6**, at 1 week of ABCD treatment, white blood cells (WBC) levels were increased significantly from baseline (*P*<0.001). After 2 weeks of treatment, WBC and neutrophil levels were increased significantly from baseline (*P*<0.05). However, there were no significant differences in other laboratory indicators, such as red blood cells (RBCs), hemoglobin (HB), platelets (PLTs), urea nitrogen, creatinine (Scr), blood kalium, and liver function before and after ABCD treatment (*P*>0.05).

**Table 6:** Comparison of laboratory indicators at 1, 2, 3 and 4 weeks with those at baseline.

Variables	Baseline	1 week			2 weeks		3 weeks		4 weeks	
		Median	*P* ^a^ value		Median	*P* ^b^ value	Median	*P* ^c^ value	Median	*P* ^d^ value
Blood routine										
WBC, (10^9/L)	2.70	8.40	0.001		7.34	0.033	4.08	0.185	1.67	0.878
RBC, (10^12/L)	0.70	2.62	0.607		2.74	0.939	2.78	0.710	2.68	0.869
HB, (g/L)	80.20	71.00	0.534		78.3	0.591	84.00	0.931	78.55	0.883
PLT, (10^9/L)	36.00	43.00	0.316		71.00	0.428	67.00	0.094	17.50	0.207
Neutrophil count, (10^9/L)	0.15	7.64	0.083		5.56	0.034	3.14	0.142	0.99	0.861
Lymphocyte count, (10^9/L)	0.22	0.62	0.073		0.89	0.130	0.59	0.368	0.44	0.704
Renal function										
BUN, (mmol/L)	5.15	5.90	0.889		5.00	0.946	4.80	0.832	5.90	0.799
Scr, (μmol/L)	38.00	53.00	0.280		51.00	0.562	56.00	0.244	52.50	0.210
Electrolyte										
K, (mmol/L)	3.69	3.94	0.864		4.11	0.838	4.33	0.425	4.19	0.438
Na, (mmol/L)	139.00	139.00	0.917		138.90	0.870	139.00	0.569	136.50	0.661
Cl, (mmol/L)	103.30	102.30	0.317		101.00	0.327	103.00	0.739	102.50	0.617
Ca, (mmol/L)	2.12	2.12	0.921		2.14	0.515	2.26	0.546	2.51	0.787
P, (mmol/L)	1.11	1.20	>0.999		1.44	0.141	1.32	0.008	1.27	0.756
Liver function										
TBIL, (μmol/L)	11.31	8.90	0.439		7.40	0.946	11.10	0.917	10.90	0.885
DBIL, (μmol/L)	9.30	5.10	0.918		5.15	0.280	5.00	0.353	7.40	0.742
IBIL, (μmol/L)	3.30	3.20	0.140		3.15	0.231	5.70	0.945	4.50	0.721
GGT, (U/L)	45.00	153.00	0.985		95.00	0.647	60.00	0.450	41.50	0.342
ALT, (U/L)	24.00	79.00	0.139		45.50	0.944	23.00	0.345	41.50	0.601
AST, (U/L)	18.50	21.00	0.941		20.00	0.344	16.00	0.169	24.50	0.148
ALP, (U/L)	101.00	145.00	0.758		117.50	0.595	93.00	0.292	82.00	0.099
TP, (g/L)	58.40	52.50	0.817		51.10	0.864	53.50	0.963	52.85	0.899
Albumin, (g/L)	30.10	33.40	0.637		31.45	0.755	35.10	0.864	33.85	0.884
Globulin, (g/L)	18.75	19.50	0.692		19.90	0.375	18.90	0.538	21.30	0.984

*P* ^a^, *P* ^b^, *P* ^c^, *P* ^d^ represented the inter-group comparison of the change in laboratory indicators from baseline at 1-4 weeks of treatment, respectively. WBC, white blood cells; RBC, red blood cells; HB, hemoglobin; PLT, platelet; BUN, blood urea nitrogen; Scr, serum creatinine; K, serum kalium; Na, serum natrium; Cl, serum chlorine; Ca, serum calcium; P, serum phosphorus; TBIL, total bilirubin; DBIL, direct bilirubin; IBIL, indirect bilirubin; GGT, glutamyltranspeptidase; ALT, alanine aminotransferase; AST, aspartate aminotransferase; ALP, alkaline phosphatase; TP, total protein.

### Drug safety analysis

ABCD treatment-related AEs were shown in **Table 7**. Most of the AEs were grade 1-2, including 6 cases of fever, 2 cases of chills, 4 cases of elevated liver function indexes and 2 cases of hypocalcemia. Grade 3-4 AEs included fever (1 case), hypokalemia (5 cases), and decreased lymphocyte count (1 case). After symptomatic treatment, those parameters in all patients returned to normal.

**Table 7:** Adverse events (AEs) related to Amphotericin B Colloidal Dispersion (ABCD) treatment.

AEs, no. (%)	Cases (N=9)	Grade	
		G 1-2	G 3-4
Fever	7 (77.78)	6 (66.67)	1 (11.11)
Hhypokalemia	5 (55.56)	0 (0.00)	5 (55.56)
Increased glutamic-pyruvic transaminase	4 (44.44)	4 (44.44)	0 (0.00)
Increased alkaline phosphatase	4 (44.44)	4 (44.44)	0 (0.00)
Increased γ glutamine transferase	3 (33.33)	3 (33.33)	0 (0.00)
Increased glutamic oxalacetic transaminase	3 (33.33)	3 (33.33)	0 (0.00)
Hypocalcemia	2 (22.22)	2 (22.22)	0 (0.00)
Chills	2 (22.22)	2 (22.22)	0 (0.00)
Increased blood bilirubin	1 (11.11)	1 (11.11)	0 (0.00)
Decreased lymphocyte count	1 (11.11)	0 (0.00)	1 (11.11)

## Discussion

To our knowledge, this is the first study of ABCD in the treatment of hematologic malignancies associated with mucormycosis in the last 10 years. Our study confirmed that ABCD showed satisfactory safety and efficacy for the treatment of mucormycosis complicated by hematologic malignancies and could be used for this application. In the present study, a total of 9 patients with mucormycosis complicated by hematologic malignancies were included, all of whom received antifungal therapy with ABCD. The overall responses of ABCD at 2 weeks, 4 weeks, and the end of treatment were 100% (9/9), 77.8% (7/9), and 77.8% (7/9), respectively. The survival rates at 4, 8, and 12 weeks were 77.8% (7/9), 66.7% (6/9), and 66.7% (6/9), respectively. The most common adverse event was an infusion reaction, followed by hypokalemia and liver insufficiency.

At present, the incidence of mucormycosis is increasing and the incidence of mucormycosis varies across countries and regions. In developed countries, hematologic malignancies have become the main cause of mucormycosis, whereas in developing countries, diabetes and ketoacidosis are still the main causes (Prakash and Chakrabarti, 2019). In our center, the main populations affected by mucormycosis are those with hematologic malignancies and those receiving hematopoietic stem cell transplantation (HSCT). To date, it is still difficult to diagnose patients with mucormycosis complicated by hematological malignancies because there are no specific serological markers. In addition, owing to the characteristics of patients with hematological malignancies, PLT numbers are often lower than 50×10^9^/L with poor coagulation functions. It is less likely for these patients to tolerate CT-guided percutaneous lung puncture or bronchoscopy biopsy to obtain histopathological results. Therefore, in this study, only 2 out of 9 cases were proven to have mucormycosis through histopathology. Moreover, previous studies have shown that RHS is a specific imaging-based manifestation of pulmonary mucormycosis (Jung et al., 2015; Bourcier et al., 2017), which is a focal circular area of ground-glass changes surrounded by ring consolidation. In addition, in a clinical study of 21 patients receiving allo-HSCT complicated by pulmonary mucormycosis, RHS were seen both at the beginning of infection and after antifungal therapy (Bao and Liu, 2022). Therefore, in our study, all patients started empirical treatment for RHS based on CT imaging before obtaining the results of etiology.

In our study, the main therapeutic drugs used to treat mucormycosis were posaconazole suspensions or tablets, AmB, and ABCD, which are also the main drugs used to treat mucormycosis in China. The revised consensus definitions of IFD from EORTC/MSGERC (Cornely et al., 2019) recommend L-AmB as the first-line antifungal therapy; However, L-AmB made in China is different from L-AmB in other countries, and there is no significant improvement in nephrotoxicity between L-AmB made in China and AmB. Therefore, before the launch of ABCD in China, the most important drugs for mucormycosis treatment were posaconazole suspensions or tablets and AmB. Of these cases, 8 were treated with a combination of AmB and posaconazole as first-line therapy. Although current relevant research results suggest that antifungal combination therapy does not improve the prognosis of mucormycosis (Kyvernitakis et al., 2016), AmB combined with triazole drugs were still used as the main treatment plan for most patients with mucormycosis in our center before ABCD was marketed (Ma et al., 2021), owing to adverse reactions to AmB. In the present study, creatinine and urea nitrogen levels returned to normal and hypokalemia was corrected after ABCD treatment. Furthermore, both the drug responses and survival rates were significantly higher than those in our previous study (Ma et al., 2021) and other studies (Hammond et al., 2011; Lanternier et al., 2012), probably because of the timely application of AmB and ABCD. Unfortunately, in this study, 2 patients (22.22%) stopped ABCD early for economic reasons. But fortunately, ABCD entered the China’s National Reimbursement Drug List (NRDL) in July 2021, so the price of ABCD is significantly lower than that previously, greatly reducing financial pressure on patients.

In this study, the median treatment duration of ABCD was 38 (13-88) days and the median cumulative dose was 5800 (2,200-16,900) mg. Previous studies have shown that ABCD is as effective as AmB for the treatment of invasive aspergillosis, but its nephrotoxicity was lower and the median time to onset of nephrotoxicity was posterior to AmB (301 days vs. 22 days) (White et al., 1997; Bowden et al., 2002). Our research also showed that even mucormycosis patients with renal injury caused by AmB could still use ABCD as an alternative or salvage treatment, and with an extension of the administration time, renal injury did not occur again, which is consistent with previous research results (Yoshida et al., 2021). In our research, the main adverse reactions related to the application of ABCD were infusion-related AEs, hypokalemia, liver function damage, and hypocalcemia. Except for the infusion-related AEs, other adverse reactions improved after symptomatic treatment. Similar to that in other studies, infusion-related AEs were common, usually most frequent during the first infusion, and the intensity and frequency decreased with subsequent administration (Le et al., 2017). In this study, 9 patients were administered promethazine and dexamethasone as pre-treatment before ABCD to prevent the infusion-related AEs. In cases of infusion-related AEs, methylprednisolone injection was used for treatment. However, 7 patients still experienced a serious infusion reaction on the first day of infusion. As expected, 6 patients had an infusion reaction only on the first day, whereas that in Case 4 lasted for nearly 20 days, necessitating pre-treatment with dexamethasone (0.5 mg) every day for 14 days. After stopping the infusion of ABCD and giving methylprednisolone, all patients’ symptoms related to infusion-related AEs were relieved, and then ABCD treatment was continued. Similarly, a previous study enumerated the infusion-related AEs of 170 patients from 21 locations around the world. In total, 1230 ABCD infusion-related AEs occurred (mean dose of 2.8 mg/kg/d), 90% of the infusion-related AEs (1105/1230) were pre-dosed, and the overall incidence rate ratio (IRR) was 12%, with that of pre-dosed cases (11%) lower than that of non-pre-dosed cases (22%). Corticosteroids are related to a reduction in the IRR (Paterson et al., 2008). Nevertheless, the incidence of infusion-related AEs in the 9 cases included in our study was significantly higher than that in previous reports, which might be due to the biased results caused by the small sample size. According to our research results, although pretreatment could only prevent some infusion-related AEs, if proper treatment is provided, related symptoms quickly disappear. Therefore, it is not necessary to stop ABCD due to an infusion reaction. Furthermore, owing to the infusion-related AEs and lack of clinical experience, not all patients were administered the targeted dose on the first day of treatment, but this was increased to the targeted dose within 2-4 days. In our experience, the infusion reaction is controllable, and thus, it is worthwhile to try to provide a therapeutic dose on the first day to achieve a rapid bactericidal effect.

There are some limitations to our study. First, this was a single-center retrospective study. Owing to the rarity of mucormycosis, the results could be biased because of the small sample size. Second, some laboratory indicator results were unavailable. Because patients with hematological malignancies have few platelets, poor coagulation function, and cannot tolerate biopsy, only 2 of the 9 cases were proven based on pathological results, whereas the rest were probable or possible cases. More larger-scale, multicenter studies of mucormycosis in the real world should be done.

In conclusion, our study confirmed that ABCD is a favorable alternative with remarkable efficacy and safety for mucormycosis complicated by hematologic malignancies.

## Data availability statement

The original contributions presented in the study are included in the article/**Supplementary Material**. Further inquiries can be directed to the corresponding author.

## Ethics statement

The studies involving human participants were reviewed and approved by the Ethics Committee of the First Affiliated Hospital of Zhengzhou University (2021-KY-0286). The patients/participants provided their written informed consent to participate in this study.

## Author contributions

Conception and Design: XM. Administrative support: XM. Materials and samples collection: JL. Data collection and collation: JL, XM. Data analysis and interpretation: JL, XM. Manuscript writing: JL, XM. All authors contributed to the article and approved the submitted version.

## References

[B15] Bao J. Liu C. (2022). Clinical manifestations of pulmonary mucormycosis in recipients of allogeneic hematopoietic stem cell transplantation: a 21-case series report and literature review Can Respir J. 2022, 1237125. doi: 10.1155/2022/1237125 35692949 PMC9184213

[B13] Bourcier J. Heudes P. M. Morio F. Gastinne T. Chevallier P. Rialland-Battisti F.. (2017). Prevalence of the reversed halo sign in neutropenic patients compared with non-neutropenic patients: data from a single-centre study involving 27 patients with pulmonary mucormycosis (2003-2016). Mycoses 60, 526–533. doi: 10.1111/myc.12624 28429890

[B21] Bowden R. Chandrasekar P. White M. H. Li X. Pietrelli L. Gurwith M.. (2002). A double-blind, randomized, controlled trial of amphotericin b colloidal dispersion versus amphotericin b for treatment of invasive aspergillosis in immunocompromised patients. Clin. Infect. Dis. 35, 359–366. doi: 10.1086/341401 12145716

[B2] Bretagne S. Sitbon K. Desnos-Ollivier M. Garcia-Hermoso D. Letscher-Bru V. Cassaing S.. (2022). Active surveillance program to increase awareness on invasive fungal diseases: the French RESSIF network (2012 to 2018). mBio 13, e0092022. doi: 10.1128/mbio.00920-22 35499498 PMC9239099

[B6] Caillot D. Legouge C. Lafon I. Ferrant E. Pagès P. B. Plocque A.. (2018). [Retrospective study of 25 cases of pulmonary mucormycosis in acute leukaemia]. Rev. Des. maladies respiratoires 35, 452–464. doi: 10.1016/j.rmr.2017.11.009 29754839

[B5] Chamilos G. Lewis R. E. Kontoyiannis D. P. (2008). Delaying amphotericin b-based frontline therapy significantly increases mortality among patients with hematologic malignancy who have zygomycosis. Clin. Infect. Dis. 47, 503–509. doi: 10.1086/590004 18611163

[B1] Cornely O. A. Alastruey-Izquierdo A. Arenz D. Chen S. C. A. Dannaoui E. Hochhegger B.. (2019). Global guideline for the diagnosis and management of mucormycosis: an initiative of the European confederation of medical mycology in cooperation with the mycoses study group education and research consortium. Lancet Infect. Dis. 19, e405–ee21. doi: 10.1016/s1473-3099(19)30312-3 31699664 PMC8559573

[B11] De Pauw B. Walsh T. J. Donnelly J. P. Stevens D. A. Edwards J. E. Calandra T.. (2008). Revised definitions of invasive fungal disease from the European organization for research and treatment of Cancer/Invasive fungal infections cooperative group and the national institute of allergy and infectious diseases mycoses study group (EORTC/MSG) consensus group. Clin. Infect. Dis. 46, 1813–1821. doi: 10.1086/588660 18462102 PMC2671227

[B18] Hammond S. P. Baden L. R. Marty F. M. (2011). Mortality in hematologic malignancy and hematopoietic stem cell transplant patients with mucormycosis, 2001 to 2009. J. Antimicrob. Chemother. 55, 5018–5021. doi: 10.1128/aac.00536-11 PMC3195043 21876046

[B8] Herbrecht R. Letscher-Bru V. Bowden R. A. Kusne S. Anaissie E. J. Graybill J. R.. (2001). Treatment of 21 cases of invasive mucormycosis with amphotericin b colloidal dispersion. Eur. J. Clin. Microbiol. Infect. Dis. 20, 460–466. doi: 10.1007/s100960100528 11561801

[B14] Jung J. Kim M. Y. Lee H. J. Park Y. S. Lee S. O. Choi S. H.. (2015). Comparison of computed tomographic findings in pulmonary mucormycosis and invasive pulmonary aspergillosis. Clin. Microbiol. Infect. 21, 684 e11–8. doi: 10.1016/j.cmi.2015.03.019 25882362

[B16] Kyvernitakis A. Torres H. A. Jiang Y. Chamilos G. Lewis R. E. Kontoyiannis D. P. (2016). Initial use of combination treatment does not impact survival of 106 patients with haematologic malignancies and mucormycosis: a propensity score analysis. Clin. Microbiol. Infect. 22, 811.e1–811.e8. doi: 10.1016/j.cmi.2016.03.029 27085727

[B19] Lanternier F. Dannaoui E. Morizot G. Elie C. Garcia-Hermoso D. Huerre M.. (2012). A global analysis of mucormycosis in France: the RetroZygo study (2005-2007). Clin. Infect. Dis. 54 Suppl 1, S35–S43. doi: 10.1093/cid/cir880 22247443

[B23] Le T. Kinh N. V. Cuc N. T. K. Tung N. L. N. Lam N. T. Thuy P. T. T.. (2017). A trial of itraconazole or amphotericin b for HIV-associated talaromycosis. New Engl. J. Med. 376, 2329–2340. doi: 10.1056/NEJMoa1613306 28614691

[B17] Ma X. Li A. Cao W. Li H. Zhang S. Li L.. (2021). Characteristics of mucormycosis in hematological patients and a death prediction model. Front. Microbiol. 12. doi: 10.3389/fmicb.2021.784974 PMC8714886 34975805

[B3] Muthu V. Agarwal R. Dhooria S. Sehgal I. S. Prasad K. T. Aggarwal A. N.. (2021). Has the mortality from pulmonary mucormycosis changed over time? a systematic review and meta-analysis. Clin. Microbiol. Infect. 27, 538-549. doi: 10.1016/j.cmi.2020.12.035 33418022

[B9] Oppenheim B. A. Herbrecht R. Kusne S. (1995). The safety and efficacy of amphotericin b colloidal dispersion in the treatment of invasive mycoses. Clin. Infect. Dis. 21, 1145–1153. doi: 10.1093/clinids/21.5.1145 8589134

[B24] Paterson D. L. David K. Mrsic M. Cetkovsky P. Weng X. H. Sterba J.. (2008). Pre-medication practices and incidence of infusion-related reactions in patients receiving AMPHOTEC: data from the patient registry of amphotericin b cholesteryl sulfate complex for injection clinical tolerability (PRoACT) registry. J. Antimicrob. Chemother. 62, 1392–1400. doi: 10.1093/jac/dkn394 18812423

[B12] Prakash H. Chakrabarti A. (2019). Global epidemiology of mucormycosis. J. Fungi (Basel) 5, 26. doi: 10.3390/jof5010026 PMC6462913 30901907

[B4] Roden M. Zaoutis T. Buchanan W. Knudsen T. Sarkisova T. Schaufele R.. (2005). Epidemiology and outcome of zygomycosis: a review of 929 reported cases. Clin. Infect. Dis. 41, 634–653. doi: 10.1086/432579 16080086

[B7] Ullmann A. J. Sanz M. A. Tramarin A. Barnes R. A. Wu W. Gerlach B. A.. (2006). Prospective study of amphotericin b formulations in immunocompromised patients in 4 European countries. Clin. Infect. Dis. 43, e29–e38. doi: 10.1086/505969 16838223

[B20] White M. H. Anaissie E. J. Kusne S. Wingard J. R. Hiemenz J. W. Cantor A.. (1997). Amphotericin b colloidal dispersion vs. amphotericin b as therapy for invasive aspergillosis. Clin. Infect. Dis. 24, 635–642. 9145737

[B10] White M. H. Bowden R. A. Sandler E. S. Graham M. L. Noskin G. A. Wingard J. R.. (1998). Randomized, double-blind clinical trial of amphotericin b colloidal dispersion vs. amphotericin b in the empirical treatment of fever and neutropenia. Clin. Infect. Dis. 27, 296–302. doi: 10.1086/514672 9709879

[B22] Yoshida M. Tamura K. Masaoka T. Nakajo E. (2021). A real-world prospective observational study on the efficacy and safety of liposomal amphotericin b in 426 patients with persistent neutropenia and fever. J. Infect. Chemother. 27, 277–283. doi: 10.1016/j.jiac.2020.10.005 33109439

