# Supplementary material for: Amphotericin B colloidal dispersion: an effective drug for the treatment of mucormycosis in China
Source: Front Cell Infect Microbiol. 2023 May 17;13:1147624. doi: 10.3389/fcimb.2023.1147624 (PMC10230081; doi:10.3389/fcimb.2023.1147624)
Supplement: Supplementary file 1 [file Table_1.docx]

**Supplementary table 1 Amphotericin B Colloidal Dispersion (ABCD) treatment in each case.**

| Cases | Mode of infusion | Time of infusion, (h) | Initial dose, (mg) | Ascending time to maximum dose, (days) | Maintenance dose, (mg) | Treatment duration, (days) | Reasons for termination of treatment |
| --- | --- | --- | --- | --- | --- | --- | --- |
| 1 | PICC | 10 | 50 | 3 | 150 | 39 | Treatment abandonment |
| 2 | PICC | 8 | 50 | 3, 31 | 150, 200 | 38 | Treatment abandonment |
| 3 | PICC | 8 | 50 | 3 | 200 | 35 | Switch to oral medication |
| 4 | PICC | 8 | 50 | 3, 7 | 150, 200 | 88 | Switch to oral medication |
| 5 | PICC | 8 | 50 | 2, 7 | 150, 200 | 13 | Treatment abandonment |
| 6 | PICC | 8 | 50 | 3, 5 | 150, 200 | 47 | Treatment abandonment |
| 7 | PICC | 8 | 50 | 4 | 150 | 18 | Treatment abandonment |
| 8 | PICC | 8 | 50 | 4 | 200 | 23 | Switch to oral medication |
| 9 | PICC | 8 | 50 | 2, 7 | 150, 200 | 44 | Switch to oral medication |

Note: none of the cases were associated with antifungal-related surgery during treatment with ABCD. Abbreviations: ABCD, Amphotericin B Colloidal Dispersion.
